# An Unpleasant Experience with Cocaine

**Published:** 1899-05

**Authors:** J. Charters Birch


### An Unpleasant Experience with Cocaine.

BY J. CHARTERS BIRCH, L.D.S.I.

Read at Meeting of Leeds and District Section of Midland Branch, December 15, 1898.

A few weeks ago I had a very unpleasant experience with cocaine, particulars of which may prove useful.

I was fitting gold collars for crowns to left mandibular cuspid and bicuspids, first trying each one to place separately, and using some little pressure to slightly cut the surrounding gum. I applied to the neck of each of the three roots, on a pellet of wool, one minim of 5 percent solution of cocaine. I am particular in saying one minim to each, for the small glass container in which I place my solution, which is freshly prepared for each case, holds ten minims, and I roll up and place in the cup at starting ten wool pellets or rolls; these absorb all the fluid.

Placing a roll of absorbent wool between the tongue and alveolus to absorb any solution that might run down with the saliva, I waited about two minutes, changing the position of the pellets occasionally. I then removed the wool and tried the collar upon the first bicuspid. Finding the gum still sensitive I applied a fresh pellet of wool to that root, leaving the others in their place. In about another minute or so I tried the collar upon the posterior root, and finding the gum insensitive, pressed the collar down to place and burnished it close; then removing the remaining pellets of wool, also the one under the tongue which was now in my way, I commenced to fit another collar. It had just got down to place without pain, when the patient remarked that her tongue and throat felt queer, as though they had no feeling, and she did not know whether she was swallowing or not; also that her hands were very cold; I felt them and they were deathlike. I then noticed that she had turned a most ghastly hue, and had dropped down helplessly in the chair, the pulse was scarcely perceptible, but hard and rapid; her breathing quick and short, and appearing to come from the throat, not from the chest at all. Removing her to the couch we laid her full length, but there she was worse and struggled to sit up as she could not breathe while extended. She breathed rather better when she sat upright propped with pillows.

Opening the case containing nitrate of amyl capsules I found four all broken, some one evidently had been trying them to find out what they contained, I therefore gave the patient a teaspoonful of sal volatil, following that with a cup of hot, strong coffee, and later with hot brandy and water.

She appeared to be in a state of collapse, hands, body and feet cold; face blanched and grey, with peculiar bluey-green color; shivering and shaking constantly. We drew the couch up to the fire, wrapped her in blankets, rubbed her hands and limbs, applied hot water, and gradually, though not for several hours, she rallied, and her breathing improved. Then her color became better, and later the pulse, and at the expiration of four hours she was able to go home in a conveyance.

After she left I examined all the appliances, and found six unused pellets, so at the most, she could only have had four minims of 5 per cent. cocaine solution applied to her gums on wool; that means perhaps $\frac{1}{5}$ gr., allowing for what remained in the wool unabsorbed.—*Journ. of the British Dental Association.*

